# Editorial: Animacy in cognition: effects, mechanisms, and theories

**DOI:** 10.3389/fpsyg.2024.1508218

**Published:** 2024-11-05

**Authors:** Michael J. Serra, Josefa N. S. Pandeirada, Joshua E. VanArsdall

**Affiliations:** ^1^Department of Medical Education, School of Medicine, Texas Tech University Health Sciences Center, Lubbock, TX, United States; ^2^William James Center for Research, Department of Education and Psychology, University of Aveiro, Aveiro, Portugal; ^3^Department of Psychology, Elmhurst University, Elmhurst, IL, United States

**Keywords:** animacy, animacy effects, animate, inanimate, cognition, perception, memory, language

## 1 Animacy in cognition

The distinction between living (animate) and non-living (inanimate) things is a crucial part of our cognition, with animate things typically receiving more attention in our thoughts and actions (Blakemore et al., 2003; Bugaiska et al., 2019; Nairne et al., 2017; Rakison and Poulin-Dubois, 2001). Beyond simply being “alive” or “not alive”, animates differ from inanimates in various ways—they can think, reproduce, move purposefully, and are perceived as being similar to humans (VanArsdall and Blunt, 2022). Living things might have driven the evolution of our cognitive processes given their greater relevance to our survival and reproduction (Nairne et al., 2013, 2017).

Our Research Topic was motivated by two main goals. First, we wanted to highlight new findings on animacy's role in cognition. While cognitive scientists have long studied animacy's influence on attention, perception, language, categories, memory, and other cognitive functions, we continue to refine our understanding of the concept and its influence. Second, we aimed to bridge researchers from various fields—cognitive psychology, linguistics, computer science, human factors, robotics, and more—to deepen our understanding of animacy's effects on our thoughts and actions. Despite varying in scope and topicality, at a higher level, the articles published in this Research Topic all focused on animacy's effects on attention, perception, memory, or language.

## 2 Articles in this Research Topic

### 2.1 Animacy, attention, and perception

Animates naturally capture our attention more than inanimates, and we often perceive animacy in non-living or artificial stimuli that display animate qualities (Rakison and Poulin-Dubois, 2001). However, Loucks et al. showed that not all animate things receive equal attention—mammals, for example, might be prioritized over insects. And though we usually think that perceiving animacy draws our attention, Saito et al. found that the reverse can also happen: we may perceive greater animacy in things that receive continued attention.

Research on animacy perception often focuses on the role of motion (Blakemore et al., 2003). Parovel reviewed how we automatically perceive animacy in simple “Heider-Simmel” animations, arguing that motion helps us identify living things and infer their psychological, emotional, and social characteristics. Torabian and Grossman discussed how children learn to see such movements as goal-directed and eventually attribute them to mental states like beliefs or desires. Animacy perception also has downstream consequences, as Mayer et al. found that people perceive anthropomorphized self-driving vehicles similarly to humans, and that humanlike qualities influence social judgments like responsibility and morality.

### 2.2 Animacy and memory

People tend to remember animate concepts better than inanimate ones (Nairne et al., 2024). While this effect is well-documented in adults, Bugaiska et al. found it occurs in older children but possibly not younger ones, likely due to their still-developing episodic memory skills. Serra and DeYoung showed that the animacy advantage in free-recall exists under both computer-paced and self-paced conditions, and that while participants' beliefs about animacy do not impact the animacy effect directly (DeYoung and Serra, 2021), they can influence processing decisions (e.g., self-paced study) and the size of the effect as a result. Mah et al. replicated Popp and Serra's (2016) finding of an *inanimate* advantage in cued-recall tasks, investigating (and ruling out) semantic similarity among animates as an explanation.

### 2.3 Animacy and language

Living things tend to take precedence over non-living things in our speech and writing (Branigan et al., 2008). Czypionka et al. examined how easily people process German noun–noun pairs and found greater processing fluency when more animate words were included (e.g., “food bowl” vs. “dog food” vs. “sheep dog”). Lobben and Laeng used Construal Level Theory to explain linguistic puzzles involving prominence hierarchies (like animacy), concluding prominent concepts are less psychologically distant from the self. Sá-Leite et al. reviewed the picture-word interference paradigm, a tool for measuring retrievability, and noted that many studies have neglected animacy despite its known enhancement of cognitive and linguistic processing. Westbury explored how people decide if something is animate or not, challenging the notion that this is a simple, binary classification (see also VanArsdall and Blunt, 2022). His analyses suggest that people rely heavily on categorical family resemblance to judge animacy.

## 3 Final thoughts

Together, the articles in this Research Topic highlight key findings and new insights on animacy's role in cognition. The articles on attention and perception not only identify factors that lead to the perception of animacy, but more uniquely how animacy affects downstream judgments and decisions that we make. The memory studies identify new conditions that augment, suppress, and even moderate the animacy advantage in memory; these are important for understanding the process(es) responsible for the effects of animacy on memory. The reasons for the prominence of animacy in language, and the downstream effects of that prioritization, are explored in the articles on language. We hope that by bringing together these diverse insights, this Research Topic deepens our understanding of how animacy influences cognition and inspires further research.
